# Prevalence and associated factors of cataract and cataract-related blindness in the Russian Ural Eye and Medical Study

**DOI:** 10.1038/s41598-020-75313-0

**Published:** 2020-10-23

**Authors:** Mukharram M. Bikbov, Gyulli M. Kazakbaeva, Timur R. Gilmanshin, Rinat M. Zainullin, Ildar F. Nuriev, Artur F. Zaynetdinov, Gulnara Z. Israfilova, Songhomitra Panda-Jonas, Inga I. Arslangareeva, Ellina M. Rakhimova, Iulia A. Rusakova, Jost B. Jonas

**Affiliations:** 1 Ufa Eye Research Institute, 90 Pushkin Street, Ufa, Bashkortostan 450077 Russia; 2 Department of Ophthalmology, Medical Faculty Mannheim of the Ruprecht-Karls-University of Heidelberg, Theodor-Kutzerufer 1, 68167 Mannheim, Germany

**Keywords:** Health care, Public health, Epidemiology

## Abstract

To assess the prevalence of cataract and cataract surgery in a population from Russia, we conducted the population-based Ural Eye and Medical Study with 5899 participants (80.5% out of 7328 eligible individuals), with an age of 40 + years as the eligibility criterion. In the phakic population, the prevalence of nuclear, cortical, subcapsular cataract and any cataract was 38.0% [95% confidence interval (CI) 36.6, 39.3], 14.5% (95% CI 13.5, 15.5), 0.6% (95% CI 0.4, 0.8) and 44.6% (95% CI 43.2, 46.0), respectively. A higher prevalence of nuclear cataract was associated with older age [odds ratio (OR) 1.10; 95% CI 1.10, 1.11], the female sex (OR 1.27; 95% CI 1.08, 1.50), urban region (OR 2.00; 95% CI 1.71, 2.33), a low educational level (OR 0.93; 95% CI 0.88, 0.98), a high diastolic blood pressure (OR 1.01; 95% CI 1.001, 1.02), a low serum concentration of high-density lipoproteins (OR 0.91; 95% CI 0.84, 0.98), more smoking package years (OR 1.01; 95% CI 1.01, 1.02), chronic kidney disease (OR 1.02; 95% CI 1.10, 1.03), a short axial length (OR 0.93; 95% CI 0.86, 0.99), and a low prevalence of age-related macular degeneration (OR 0.72; 95% CI 0.57, 0.92). The prevalence of previous cataract surgery conducted in 354/5885 individuals (6.0%; 95% CI 5.4, 6.6) increased from 0.4% (95% CI 0.0, 1.0) in the age group of 40–45 years to 37.6% (95% CI 30.9, 44.4) in the age group of 80 + years. Cataract was the cause of moderate-to-severe vision impairment in 109 (1.8%) individuals and of blindness in three (0.05%) individuals. The prevalence of cataract and cataract-related MSVI and blindness were relatively high; subsequently, the prevalence of previous cataract surgery was relatively low in this population from Russia.

## Introduction

Cataract is the most common cause of blindness and, together with undercorrection of refractive error, is the most common cause of moderate and severe vision impairment^1,2^. The increase in the sociodemographic index of many low-to-medium income countries and the improvement of surgical techniques to treat cataract have led to an increase in the cataract surgical rate in the last 2 decades. This was associated with a decrease in the percentage of low vision and blindness caused by cataract^1–3^. No detailed information has been available regarding the prevalence of cataract surgery and its outcomes in Russia nor the prevalence of the various forms of cataract and their associations with ocular and systemic parameters. Furthermore, since Russia is by surface the largest and one of the most populous countries worldwide, we conducted this population-based study in Russia to assess the prevalence of cataract and its various forms, the frequency of cataract-related blindness and vision impairment, and the prevalence of cataract surgery in Russia.

## Methods

The population-based Ural Eye and Medical Study was conducted in the city of Ufa in the district of Kirovskii and in villages in the rural region of the Karmaskalinsky District 65 km from Ufa^4–6^. The study period was from 2015 to 2017. The Ethics Committee of the Academic Council of the Ufa Eye Research Institute approved the study and confirmed that all methods were performed in accordance with the relevant guidelines and regulations, and all participants gave written informed consent. Ufa is the capital of the Republic of Bashkortostan in the Volga Federal District and is the economic, scientific and cultural center of the region. Situated 1300 km east of Moscow in the west of the Southern Ural mountains, Ufa has 1.1 million inhabitants. The population of the republic of Bashkortostan (population: 4.07 million; area: 143,600 km^2^) includes Russians (36.1%), Tatars (25.4%), Bashkirs (29.5%), Chuvash (2.7%), Mari (2.6%), Ukrainians (1.0%) and other ethnicities. From a climatic point of view, the hottest month is July, with an average high temperature of 25.9 °C, and the coldest months are January and February, with average low temperatures of − 17 °C. All people residing in the study regions were officially registered, and home visits were performed according to their registration. Eligible subjects fulfilling the inclusion criterion of aged 40 + years were visited up to three times if they did not participate in the study after the first visit. The only inclusion criteria for the study were living in the study region and an age of 40 + years. There were no exclusion criteria.

The examinations started with an interview consisting of 256 standardized questions on socioeconomic parameters, such as level of education; family income and family possessions; living conditions (such as a toilet available in the house, lighting source, agricultural land and livestock ownership, size of family); diet (such as frequency and amount of intake of vegetables, fruits and meat); smoking or other types of tobacco consumption; daily physical activity; alcohol consumption; presence of ocular problems; availability of an ophthalmologist; availability and wearing of glasses; depression and suicidal thoughts; medical history, including known diagnosis and therapy of arterial hypertension, diabetes mellitus, angina, asthma and other pulmonary problems, cardiovascular and cerebrovascular diseases, lower back pain, malignancies, menstruation and related issues, previous trauma including bone fractures, and hearing problems. The questions were taken from standardized questionnaires, such as the Zung self-rated depression scale and the Mini Mental Status Examination test^7,8^. The level of education was categorized into the stages of “illiteracy” (no reading ability at all), “passing of the 5th grade”, “passing of the 8th grade”, “passing of the 10th grade”, “passing of the 11th grade”, “graduation”, and “postgraduation”. The questions additionally included standardized questions on the amount and frequency of smoking and alcohol consumption and living conditions and were previously included and tested in other population-based studies, such as the Central India Eye and Medical Study and the Beijing Eye Study^9,10^. The interview was conducted by trained social workers who personally asked the questions and filled the answers into the questionnaire.

Medical examinations included measurement of blood pressure, handgrip force and anthropometric parameters. We conducted a spirometric test for the assessment of chronic obstructive pulmonary disease and a biochemical analysis of blood samples taken under fasting conditions. The ophthalmologic examinations included automatic and subjective refractometry for determination of best corrected visual acuity (BCVA), perimetry (PTS 1000 Perimeter, Optopol Technology Co., Zawercie, Poland), anterior segment biometry (Pentacam HR, Typ70900, OCULUS, Optikgeräte GmbH Co., Wetzlar, Germany), slit lamp biomicroscopy of the anterior ocular segment, and noncontact tonometry (Tonometer Kowa KT-800, Kowa Company Ltd., Hamamatsu City, Japan). After medically inducing mydriasis (tropicamide 0.8% and phenylephrine 5% given twice in a 10-min interval), a second slit lamp examination was performed by a board-certified ophthalmologist to assess the presence of pseudoexfoliation of the lens^11^. Pseudoexfoliation was differentiated into seven grades or stages, with stage 0 for “no pseudoexfoliation”, stage 1 for “faint pseudoexfoliation” (small dark islands in the intermediary annular region corresponding to the moving pupillary margin), stage 2 for “confluent dark islands in the annular region”, stage 3 for “visible edges of pseudoexfoliative material clearly detectable in at least one location on the lens surface”, stage 4 for “complete circular edge of pseudoexfoliative material on the lens surface (central island or in the lens periphery)”, stage 5 for “pseudoexfoliative dandruff on the pupil margin”, and stage 6 for “pseudoexfoliative material on the corneal endothelium, in the anterior chamber angle, and/or lens subluxation”. A similar grading classification was described by Prince and associates^12^. Using the slit lamp, digital photographs of the cornea and lens were taken (Topcon slit lamp and camera, Topcon Corp. Tokyo, Japan). For the lens photographs, the slit lamp beam had a width of 0.3 mm and a height of 9.0 mm. The angle between the slit lamp beam and the sagittal axis was 45°. The slit lamp beam bisected the central lens from its superior pole at the 12:00 position to its inferior pole at the 6:00 o’clock position. The beam was focused onto the center of the lens nucleus. Additionally, retro-illuminated photographs of the lens were taken.

Comparing the lens photographs with seven standard photographs of lenses with increasingly severe nuclear opacities, the presence and degree of cataract were assessed by applying the scheme of the Age-Related Eye Disease Study^13^. Nuclear cataract was divided into six grades. We combined standard photograph 6 and 7 into one grade. We defined the presence of nuclear cataract as a nuclear cataract grade of 3 or higher. The degrees of cortical lens opacification and posterior subcapsular lens opacification were assessed using photographs taken by retro-illumination (Topcon slit lamp and camera, Topcon Corp. Tokyo, Japan). Cortical and posterior subcapsular opacities appeared as darkly shaded interruptions of the reddish-orange fundus reflex on these photographs. Any lens area that was definitely darkened was considered involved, regardless of the density of the opacity. Using a grid, the degrees of cortical cataract and subcapsular cataract were measured as the percentage areas of opacity. The presence of cortical cataract and subcapsular cataract was defined by the presence of any cortical or subcapsular opacity, respectively.

We additionally took photographs of the optic nerve head and macula (VISUCAM 500, Carl Zeiss Meditec AG, Jena, Germany), and spectral-domain optical coherence tomographic (OCT) images were obtained (RS-3000, NIDEK co., Ltd., Aichi Japan). The latter served to measure the peripapillary retinal nerve fiber layer thickness, neuroretinal rim width, and thickness of the retina. The degree of fundus tessellation was examined on the fundus photographs^14^. We defined age-related macular degeneration (AMD) as suggested by the recent Beckman Initiative for Macular Research Classification Committee^15^. For the definition of glaucoma, we applied criteria recommended by the ISGEO (International Society of Geographical and Epidemiological Ophthalmology)^16^. All examinations and the interview were conducted in the Ufa Eye Research Institute in Ufa. We applied the Guidelines for Accurate and Transparent Health Estimates Reporting (GATHER statement guidelines) for collecting the data^17^. All medical and ophthalmological examinations were performed by specially trained technicians with daily supervision by medical doctors and short-term statistical evaluation of the measured data. The images were primarily examined by trained ophthalmologists supervised by staff members of the institute. All clinical images with an unclear diagnosis, all images of eyes with an elevated intraocular pressure, any abnormality of the macula or optic nerve head, and any best corrected visual acuity of equal to or worse than 0.20 logMAR (Snellen 6/9.5, decimal: 0.63) were reassessed by a panel (including MMB and JBJ).

Diagnostic criteria for diabetes mellitus were a fasting serum glucose concentration of ≥ 7.0 mmol/L or a self-reported history of physician-based diagnosis or therapy of diabetes mellitus. Inclusion criteria for the current investigation were the availability of information of the phakic/pseudophakic/aphakic status of the eye as assessed by slit lamp biomicroscopy and the availability of photographs of the lens for the assessment of the type and amount of cataract. We analyzed the data statistically using a statistical software package (SPSS for Windows, version 25.0, IBM-SPSS, Chicago, IL, USA). In the first step, we calculated the mean prevalence of cataract and pseudophakia [expressed as the means and 95% confidence intervals (CI)]. A participant was considered to have undergone cataract surgery if the surgery had been performed on one of both eyes, or on both eyes, of an individual. For the prevalence of the various cataract forms, data from a randomly selected eye per study participant were taken for the statistical analysis. As the second step, we performed a binary univariate regression analysis of associations between the prevalence of pseudophakia/aphakia or the prevalence of the various forms of cataract and other ocular and systematic parameters. This was followed by a multivariable binary regression analysis, in which the prevalence of pseudophakia/aphakia or the prevalence of the various cataract forms were the dependent variable, and the independent parameters were variables that were significantly (*P* ≤ 0.05) associated with the prevalence of pseudophakia/aphakia or the prevalence of the various cataract forms in the univariate analyses. In a step-by-step manner, we removed variables from the list of independent parameters that either showed a high collinearity or that were no longer significantly associated with the prevalence of pseudophakia/aphakia or the prevalence of the various cataract forms. In the case of collinearity, we usually removed the parameter with the higher variance inflation factor. We eventually performed a Bonferroni correction of the associations found in the multivariate analysis by multiplying the *P* values with the number of independent parameters in the final multivariate model. We determined the odds ratios (OR) and their 95% CI. Only one eye per study participant was included in the statistical analysis. All *P* values were two-sided and considered statistically significant when the values were less than 0.05.

## Results

Out of a population of 7328 eligible individuals, 5899 (80.5%) individuals [2580 (43.7%) men] participated (mean age: 59.0 ± 10.7 years [range 40–94 years)] in the Ural Eye and Medical Study. There were 1185 (22.0%) Russians, 2439 (45.2%) Tartars, 1059 (19.6%) Bashkirs, 587 (10.9%) Chuvash, 21 (0.4%) Mari, and 104 (1.9%) individuals of other ethnicities, while 504 participants did not report their ethnicity. According to the census carried out in Russia in 2010, the composition of the population of the Ural Eye and Medical Study with respect to sex and age corresponded to the sex and age distribution in the Russian population beyond an age of 40 + years, with two constrictions in both populations due to the consequences of World War II^18,19^. With respect to the ethnic composition, the study population compared to the total population of Russia had a markedly higher proportion of Tartars (3.7% in whole Russia) and Bashkirs (1.1% in whole Russia), and correspondingly, a lower proportion of Russians (77.7% in whole Russia).

Information about the surgical lens status (phakia/pseudophakia/aphakia) was available for 5885 (99.8%) individuals, with a mean age of 59.0 ± 10.7 years (range 40–94 years) and a mean axial length of 23.3 ± 1.1 mm (range 19.78–32.87 mm). The assessment of the natural lens based on lens photographs was performed for 5100 (86.5%) phakic individuals. There was a lower number of individuals with assessment of the natural lens status compared to the total number of study participants because of the exclusion of eyes after cataract surgery from the group of eyes with lens assessment [n = 354 (354/5885 or 6.0%) individuals] and because lens photographs were not taken for the first 445 (445/5899 or 7.6%) phakic individuals at the start of the study. The group of phakic individuals with assessment of the lens status compared with the group of individuals without assessment of the lens status was significantly (*P* < 0.001) younger (57.9 ± 10.1 years versus 65.6 ± 11.8 years), showed a significantly (*P* < 0.001) lower proportion of women (2803/5100 or 55.0% versus 516/799 or 64.6%), and did not differ significantly (*P* = 0.82) in axial length (23.3 ± 1.1 mm versus 23.3 ± 1.3 mm).

**Figure 1:**
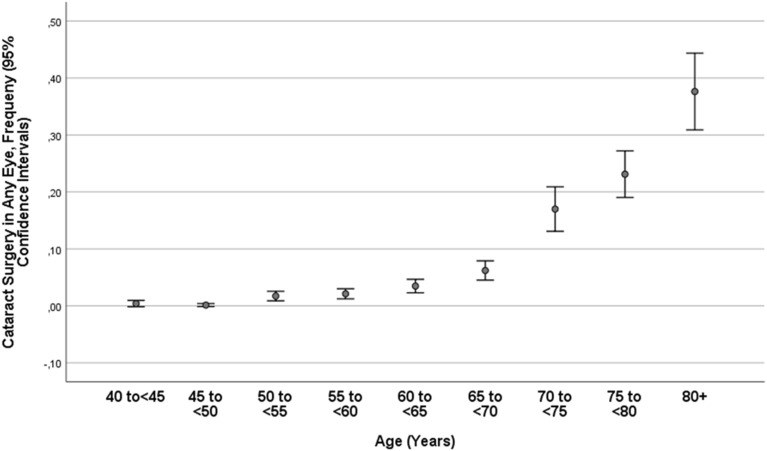
Prevalence of cataract surgery in the Ural Eye and Medical Study.

Cataract surgery in one or both eyes of the same participant had been performed in 354/5885 individuals (6.0%; 95% CI 5.4, 6.6) (Table 1). Among the 497 eyes that had undergone cataract surgery, 28 (5.8%) were aphakic, 8 (1.6%) had an anterior chamber intraocular lens, and the remaining 461 (92.8%) had a posterior chamber intraocular lens. The prevalence of previous cataract surgery increased from 2/495 (0.4%) in the age group of 40–< 45 years to 32/920 (3.5%) in the age group of 60–< 65 years to 76/202 (37.6%) in the age group of 80 + years (Table 1; Fig. 1). After adjusting for age, a higher prevalence of cataract surgery associated (*P* < 0.05) with the systemic parameters of male sex, urban region of habitation, ethnicity (Russian versus non-Russian), taller body height, higher prevalence of a positive history of bone fracture, heart attack and steroid (cortisone) therapy, and a higher State-Trait Anxiety Inventory (STAI) score. A higher prevalence of cataract surgery was also correlated (*P* < 0.05) with the ocular parameters of longer axial length, more myopic refractive error, deeper anterior chamber depth, larger anterior chamber volume, wider anterior chamber angle, lower retinal nerve fiber layer thickness, lower prevalence of pseudoexfoliation of the lens, higher prevalence and stage of glaucoma and higher prevalence of open-angle glaucoma, higher prevalence and stage of diabetic retinopathy, higher prevalence and stage of myopic maculopathy, and higher best corrected visual acuity.

**Table 1:** Prevalence (mean and 95% confidence interval) and mean stage of nuclear cataract, cortical cataract, subcapsular cataract and any cataract and status after cataract surgery stratified by age in the Ural Eye and Medical Study.

Age group (years)	n	Nuclear cataract	Cortical cataract	Subcapsular posterior cataract	Any cataract	n	Cataract Surgery
40–< 45	453	9.5% (6.8, 12.29)	3.8% (2.0, 5.5)	0.2% (0.0, 0.7)	13.4 (10.3, 16.6)	495	0.4% (0.0, 1.0)
45–< 50	703	16.5% (13.8, 19.3)	4.6% (3.0, 6.1)	0.1% (0.0, 0.4)	20.6% (17.6, 23.6)	742	1.4% (0.0, 0.4)
50–< 55	869	23.5% (20.7, 26.3)	6.3% (4.7, 8.0)	0.6% (0.1, 1.1)	28.4% (25.4, 31.4)	928	1.7% (0.9, 2.6)
55–< 60	946	32.9% (30.0, 35.9)	9.8% (7.9, 11.7)	0.5% (0.1, 1.0)	39.7%(36.6, 42.9)	1039	2.1% (1.2, 3.0)
60–< 65	803	41.7% (38.3, 45.1)	15.2% (12.7, 17.7)	0.6% (0.1, 1.2)	51.8% (48.3, 55.3)	920	3.5% (2.3, 4.7)
65–< 70	661	61.3% (57.6, 65.0)	25.1% (21.8, 28.4)	0.9% (0.2, 1.6)	69.7% (66.2, 73.3)	789	6.2% (4.5, 7.9)
70–< 75	268	69.0% (63.5, 74.6)	30.2% (24.7, 35.8)	1.1% (0.0, 2.4)	77.6% (72.6, 82.6)	359	17.0% (13.1, 20.9)
75–< 80	283	82.3% (77.9, 86.8)	42.8% (77.9, 86.8)	1.4% (0.0, 2.8)	88.7% (85.0, 92.4)	411	23.1% (19.0, 27.2)
80 +	114	91.2% (86.0, 96.5)	46.5% (37.2, 55.8)	0.9% (0.0, 2.6)	96.5% (93.1, 99.9)	202	37.6% (30.9, 44.4)
Total	5100	38.0% (36.6, 39.3)	14.5% (13.5, 15.5)	0.6% (0.4, 0.8)	44.6% (43.2, 46.0)	5885	6.0% (5.4, 6.6)

The prevalence of previous cataract surgery was not significantly associated with the systemic parameters of the level of education (*P* = 0.25); physical activity score (*P* = 0.15); family status (married versus unmarried; *P* = 0.84); religion (Muslim versus non-Muslim; *P* = 0.28); body weight (*P* = 0.51); body mass index (*P* = 0.15); waist-hip ratio (*P* = 0.28); self-reported family income (*P* = 0.66); history of angina pectoris (*P* = 0.40); arthritis (*P* = 0.15); cancer (*P* = 0.20); cardiovascular diseases, including stroke (*P* = 0.59); dementia (*P* = 0.35); diarrhea (*P* = 0.99); iron-deficiency anemia (*P* = 0.80); episodes with low blood pressure and hospitalization (*P* = 0.75); osteoarthritis (*P* = 0.39); injuries other than bone fracture (*P* = 0.53); other noncommunicable diseases (*P* = 0.97); backache (*P* = 0.11); headache (*P* = 0.57); neck pain (*P* = 0.16); thoracic spine pain (*P* = 0.50); skin disease (*P* = 0.37); thyreopathy (*P* = 0.38); falls (*P* = 0.92); episodes of unconsciousness (*P* = 0.27); menopause (age of any last bleeding: *P* = 0.77; age of last regular bleeding: *P* = 0.74); serum concentrations of alanine aminotransferase (*P* = 0.87), aspartate aminotransferase *P* = 0.46), bilirubin (*P* = 0.53), high-density lipoproteins (*P* = 0.95), low-density lipoproteins (*P* = 0.16), triglycerides (*P* = 0.26), cholesterol (*P* = 0.06), rheumatoid factor (*P* = 0.87), glucose (*P* = 0.61), creatinine (*P* = 0.25), urea (*P* = 0.51), residual nitrogen (*P* = 0.50) and hemoglobin (*P* = 0.25); the erythrocyte sedimentation rate (*P* = 0.41); the INR (international normalization ratio) (*P* = 0.84); prothrombin index (*P* = 0.99); erythrocyte count (*P* = 0.95); leukocyte count (*P* = 0.35); lymphocyte count (*P* = 0.15); intake of blood lipid lowering medication (*P* = 0.62); prevalence of diabetes mellitus (*P* = 0.19); anemia (*P* = 0.80); arterial hypertension (*P* = 0.43); stage of arterial hypertension (*P* = 0.92); amount of food containing whole grain (*P* = 0.54); amount of self-reported salt intake (*P* = 0.90); the grade of processing of meat (weak/medium/well done) (*P* = 0.16); systolic (*P* = 0.95), diastolic (*P* = 0.76), and mean (*P* = 0.95) blood pressure; ankle-brachial index, right side (*P* = 0.57); diet (vegetarian or mixed diet) (*P* = 0.09); number of meals taken (*P* = 0.73); number of days per week with intake of fruits (*P* = 0.20) or vegetables (*P* = 0.42); current smoker (*P* = 0.11); daily smoking (*P* = 0.14); smoked package years (*P* = 0.20); any alcohol consumed (*P* = 0.52); hearing loss total score (*P* = 0.26); depression score (*P* = 0.12); or manual dynamometry, right hand (*P* = 0.37); nor with the ocular parameters of corneal refractive power (*P* = 0.11), central corneal thickness (*P* = 0.69), lens thickness (*P* = 0.24), intraocular pressure (*P* = 0.40), retinal thickness (total) in the fovea (*P* = 0.21), 300 µm temporal to the fovea (*P* = 0.14) and 300 µm nasal to the fovea (*P* = 0.63), or prevalence of angle-closure glaucoma higher prevalence (*P* = 0.99).

In the multivariable analysis, the parameters of body height (*P* = 0.82), prevalence of myopic maculopathy (*P* = 0.80), history of heart attack (*P* = 0.66) and bone fracture (*P* = 0.51), region of habitation (*P* = 0.63), anxiety score (*P* = 0.49), prevalence of open-angle glaucoma (*P* = 0.53), history of steroid intake (*P* = 0.69). ethnicity (*P* = 0.49), prevalence of pseudoexfoliation (*P* = 0.37), retinal nerve fiber layer thickness (*P* = 0.26), sex (*P* = 0.70), and anterior chamber depth (*P* = 0.08) were removed due to the collinearity of the parameters of refractive error and anterior chamber angle and volume and due to a lack of statistical significance. In the final model, a higher prevalence of previous cataract surgery was associated with older age (OR 1.17; 95% CI 1.15, 1.19; *P* < 0.001), longer axial length (OR 1.68; 95% CI 1.47, 1.91; *P* < 0.001), and higher prevalence of diabetic retinopathy (OR 2.79; 95% CI 1.37, 5.71; *P* = 0.005).

**Figure 2:**
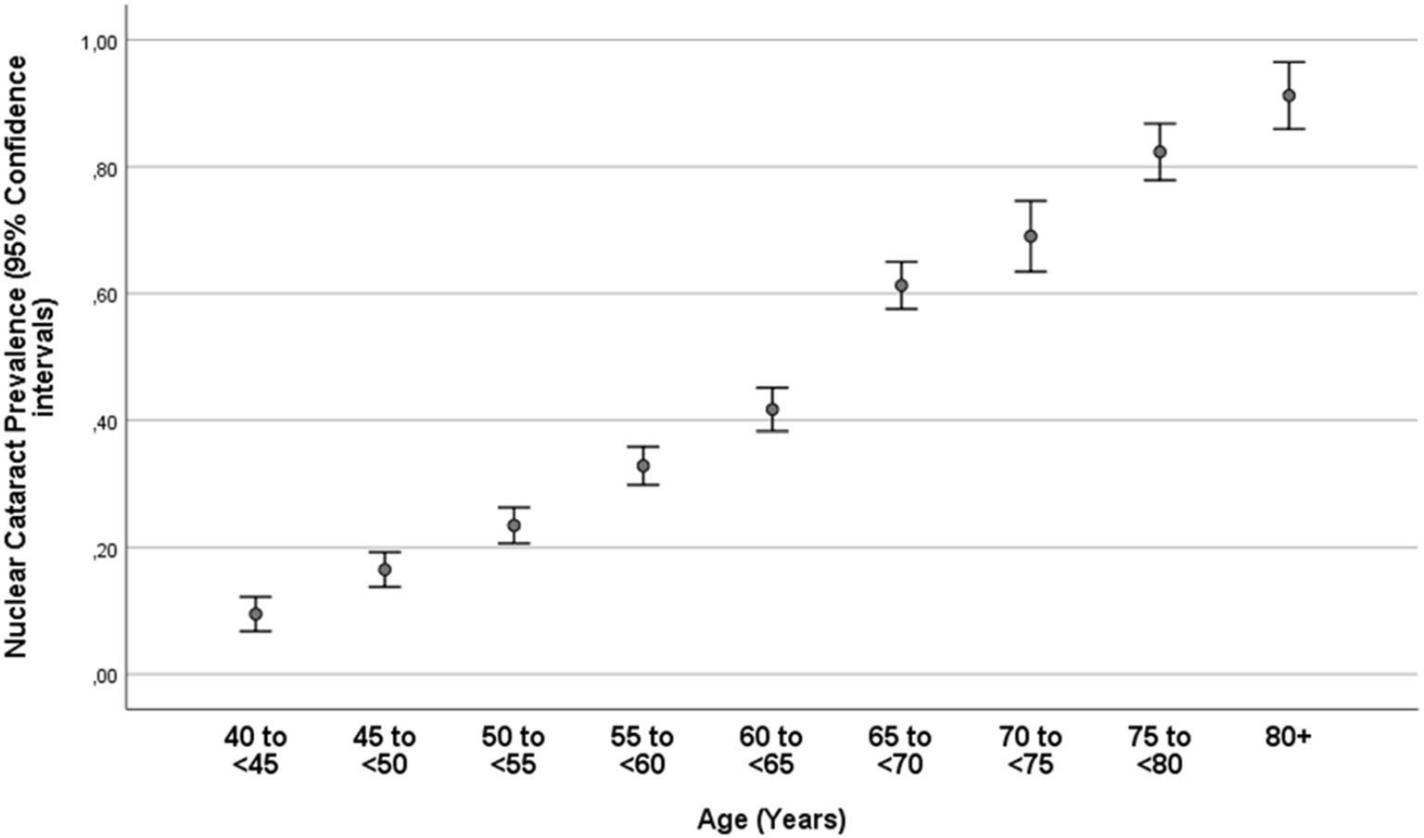
Prevalence of nuclear cataract in the phakic population of the Ural Eye and Medical Study.

**Figure 3:**
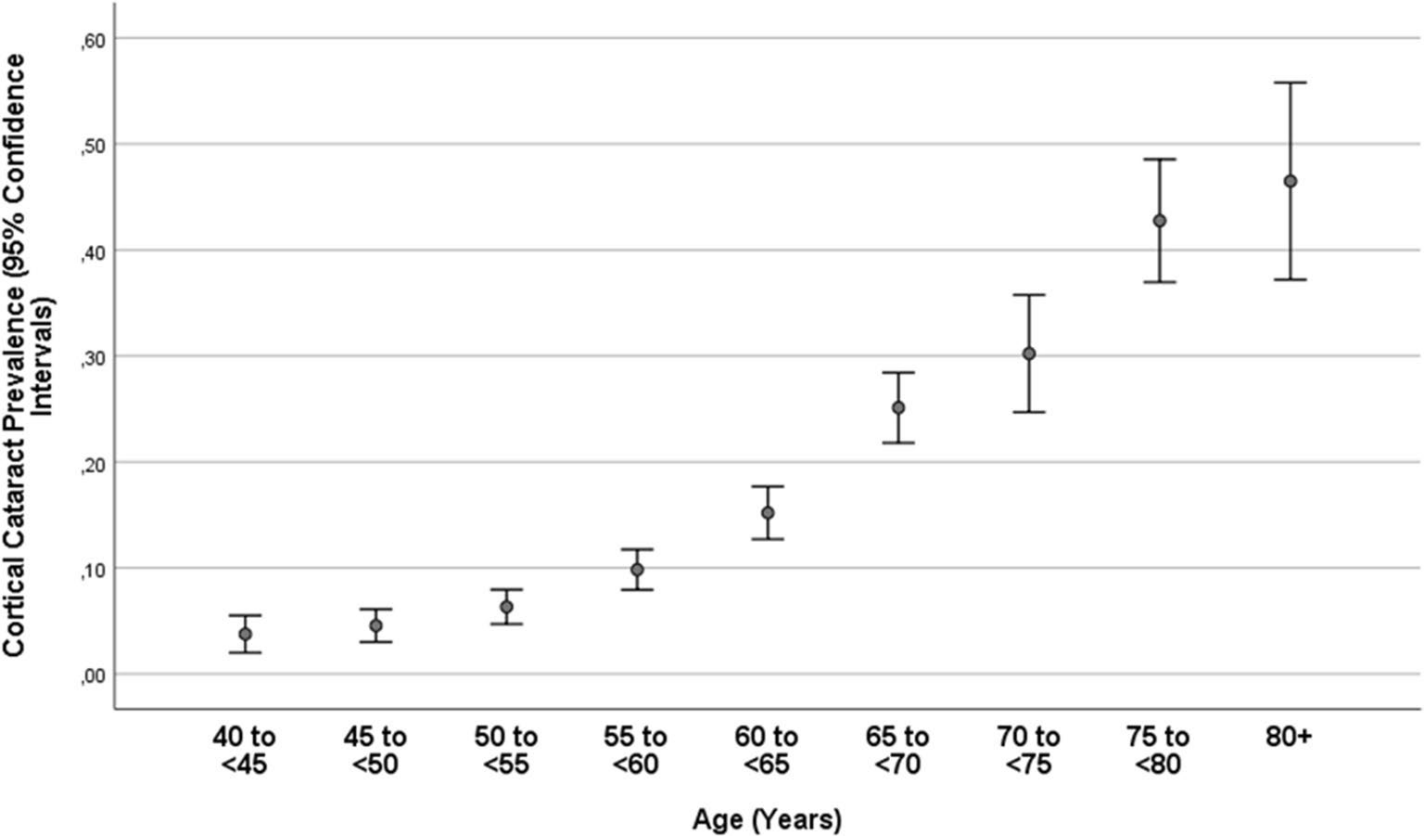
Prevalence of cortical cataract in the phakic population of the Ural Eye and Medical Study.

Nuclear cataract, cortical cataract, subcapsular posterior cataract and any cataract, as assessed in a randomly selected eye per phakic study participant, were detected in 1936 individuals (38.0%; 95% CI 36.6, 39.3), 740 individuals (14.5%; 95% CI 13.5, 15.5), 31 individuals (0.6%; 95% CI 0.4, 0.8) and 2275 individuals (44.6%; 95% CI 43.2, 46.0), respectively (Table 1). The prevalence of any cataract increased from 13.5% (95% CI 10.3, 16.6) in the age group of 40–< 45 years, to 51.8% (95% CI 48.3, 55.3) in the age group of 60–< 65 years, to 96.5% (95% CI 93.1, 99.9) in the age group of 80 + years (Table 1; Figs. 2, 3).

In the multivariable binary regression analysis, the prevalence of nuclear cataract was associated with older age, female sex, urban region of habitation, lower level of high-density lipoproteins, higher diastolic blood pressure, higher prevalence of chronic kidney disease (or higher stage of kidney disease), lower level of education, higher number of smoking package years, lower prevalence of age-related macular degeneration, and shorter axial length (Table 2). In that model, the prevalence of nuclear cataract was not significantly associated with the prevalence of diabetes (*P* = 0.61), chronic obstructive pulmonary disease (*P* = 0.76), anemia (*P* = 0.44), inflammatory parameters such as the erythrocyte sedimentation rate (*P* = 0.58), physical activity score (*P* = 0.29), any alcohol consumption (*P* = 0.88), depression score (*P* = 0.90) and degree of macular fundus tessellation (*P* = 0.70). Since eyes after surgery of previous cataract were excluded from the analysis and since cataract surgery was strongly associated with older age, we performed a second analysis in which individuals with an age of 75 + years were excluded. It showed a similar result: a higher prevalence of nuclear cataract was associated with older age, female sex, urban region of habitation, lower level of high-density lipoproteins, higher diastolic blood pressure, higher number smoking package years, and lower prevalence of age-related macular degeneration, while a lower level of education (*P* = 0.050) and shorter axial length (*P* = 0.67) were no longer significantly correlated. If in a third analysis previous cataract surgery was counted as nuclear cataract, the same results were obtained as if eyes were excluded that had undergone cataract surgery.

**Table 2:** Associations (multivariable analysis) between the prevalence of nuclear cataract and systemic and ocular parameters in the phakic population of the Ural Eye and Medical Study.

Parameter	*P* value	Odds ratio	95% confidence interval
Age (years)	< 0.001	1.10	1.10, 1.11
Male/female sex	0.004	1.27	1.08, 1.50
Urban region of habitation	< 0.001	2.00	1.71, 2.33
Serum concentration of high-density lipoproteins (mmol/L)	0.02	0.91	0.84, 0.98
Diastolic blood pressure (mmHg)	0.02	1.01	1.001, 1.02
Level of education	0.006	0.93	0.88, 0.98
Chronic kidney disease stage	0.002	1.02	1.01, 1.03
Smoking package years	< 0.001	1.01	1.01, 1.02
Age-related macular degeneration, prevalence	0.006	0.72	0.57, 0.92
Axial length (mm)	0.03	0.93	0.86, 0.99

A higher prevalence of cortical cataract was associated with older age, female sex, urban region of habitation, lower level of high-density lipoproteins, and lower prevalence of age-related macular degeneration, while the parameters of chronic kidney disease stage (*P* = 0.91), smoking package years (*P* = 0.55), diastolic blood pressure (*P* = 0.99), level of education (*P* = 0.07) and axial length (*P* = 0.13) were not significantly associated (Table 3).

**Table 3:** Associations (multivariable analysis) between the prevalence of cortical cataract and systemic and ocular parameters in the phakic population of the Ural Eye and Medical Study.

Parameter	*P* value	Odds ratio	95% confidence interval
Age (years)	< 0.001	1.10	1.09, 1.11
Male/female sex	< 0.001	1.44	1.19, 1,74
Urban region of habitation	< 0.001	0.49	0.39, 0.60
Serum concentration of high-density lipoproteins (mmol/L)	0.002	0.84	0.75, 0.94
Age-related macular degeneration, prevalence	0.01	1.37	1.07, 1.76

The prevalence of subcapsular posterior cataract was associated only with older age (OR 1.05; 95% CI 1.01, 1.08; *P* < 0.001).

The prevalence of nuclear cataract (assessed in the eye with worse nuclear cataract per phakic individual), cortical cataract (assessed in the eye with worse cortical cataract per phakic individual), subcapsular posterior cataract (assessed in the eye with worse subcapsular posterior cataract per phakic individual), and any cataract (assessed in the eye with worse cataract per phakic individual), was 40.7% (95% CI 39.4, 42.1), 19.8% (95% CI 18.7, 20.9), 1.0% (95% CI 0.7, 1.3), and 49.7% (95% CI 48.3, 51.1), respectively. The prevalence of nuclear cataract (assessed in the eye with worse nuclear cataract per phakic individual), cortical cataract (assessed in the eye with worse cortical cataract per phakic individual), subcapsular posterior cataract (assessed in the eye with worse subcapsular posterior cataract per phakic individual), and any cataract (assessed in the eye with worse cataract per phakic individual), including the pseudophakic individuals, was 36.4% (95% CI 35.2, 37.7), 17.6% (95% CI 16.6, 18.6), 0.9% (95% CI 0.7, 1.1), and 44.4% (95% CI 43.1, 545.7), respectively.

Cataract of any form as a cause of moderate to severe vision impairment (MSVI) (defined as BCVA < 6/18 but ≥ 3/60 inclusive in the better eye or in binocular viewing) was present in 109 individuals (1.8%), with mostly nuclear cataract present in 24 individuals (0.4%), mostly cortical cataract in 29 participants (0.5%), cortico-nuclear cataract in 53 individuals (0.9%), subcapsular posterior cataract in one individual (0.02%), and mature cataract in one participant (0.02%). Cataract was the cause of blindness (BCVA < 3/60) in three individuals (0.05%). Cataract was the cause of MSVI in 59.9% (95% CI 52.7, 67.1) of the 182 study participants with MSVI, and it was the cause of blindness in 27.3% of the 11 blind individuals.

The mean BCVA after cataract surgery in right eyes was 0.45 ± 0.98 logMAR (logarithm of the minimal angle of resolution) [median: 0.20 (Snellen equivalent: 20/32; decimal system: 0.63); range light perception to − 0.20 logMAR (Snellen equivalent: 20/12.5; decimal: 1.60)]. Causes of postoperative reduction of BCVA to counting fingers (at a distance of 1 m) or lower were glaucoma (5 eyes), corneal opacities (3 eyes), myopic macular degeneration (2 eyes), late stage age-related macular degeneration (2 eyes), and secondary cataract in one eye. The mean BCVA after cataract surgery in left eyes was 0.45 ± 0.97 logMAR [median 0.20 (Snellen equivalent: 20/32; decimal system: 0.63); range light perception to -0.10 logMAR (Snellen equivalent: 6/4.8; decimal system: 1.25)]. Causes of postoperative reduction of BCVA to counting fingers (at a distance of one meter) or lower were glaucoma (4 eyes), myopic macular degeneration (3 eyes), retinal detachment (2 eyes), secondary cataract (2 eyes), late stage age-related macular degeneration (1 eye), and corneal opacities (1 eye).

## Discussion

In our study of a Russian population aged 40 + years, nuclear cataract, cortical cataract, subcapsular posterior cataract and any cataract, as assessed in a randomly selected eye per phakic study participant, had a prevalence of 38.0%, 14.5%, 0.6%, and 44.6%, respectively. The prevalence of any cataract was 51.8% in phakic individuals aged 60 to < 65 years, which increased to 96.5% in phakic individuals aged 80 + years. The prevalence of cataract of any form as a cause of moderate to severe vision impairment was 1.8%; as cause of blindness, the prevalence was 0.05%. Cataract was the cause of MSVI in 59.9% of the study participants with MSVI, and it was the cause of blindness in 27.3% of the blind individuals. The prevalence of previous cataract surgery in any eye was 6.0%, increasing from 0.4% in the age group of 40–< 45 years and to 37.6% in the age group of 80 + years.

The prevalence of cataract and the frequency of cataract as a cause of vision impairment and blindness, as found in our population from Russia, were higher than in populations examined in previous population-based studies from high-income countries and medium-income countries. A meta-analysis performed by the Vision Loss Expert Group revealed that the percentage of MSVI caused by cataract was higher in our study population from Russia (59.9%) than in the high-income regions of North America (14.4%), Western Europe (15.5%), Eastern Europe (14.8%), Central Europe (18.2%) and Australasia (14.10%)^13^. The percentage observed in our study was also higher than in South and Central Asia (23.62%) and in Sub-Saharan Africa (30.8%)^14,15^. In a similar manner, the percentage of cataract-related blindness was higher in our study population (27.3%) than in studies form the high-income regions of North America (20.1%), Western Europe (21.4%), Eastern Europe (20.9%), Central Europe (25.4%), and Australasia (19.7%), while it was lower than study populations from South and Central Asia (36.6%) and Sub-Sahara Africa (41.6%)^20–22^.

Corresponding to the relatively high prevalence of cataract in general, and corresponding to the high prevalence of cataract as a cause of MSVI and blindness in particular, the prevalence of eyes with previous cataract surgery was relatively low in our study population, with a figure of 6.0% of the total study population and 37.6% of the population aged 80 + years. These figures did not differ significantly from the prevalence of previous cataract surgery in the population of the Central India Eye and Medical Study that was conducted in a rural region of Central India close to the Indian Tribal Belt from 2006 to 2008. In that study, the prevalence of previous cataract surgery was 6.5% (95% CI 5.7, 7.3) in the study population aged 40 + years, and it was 28.8% (95% CI 18.1, 39.4) in those aged 80 + years ^23^. Factors associated with a higher prevalence of previous cataract surgery in our study population were longer axial length and a higher prevalence of diabetic retinopathy after adjusting for older age. These associations may be due to a higher frequency of ophthalmological examinations of myopic patients and diabetic patients.

A relatively high prevalence of nuclear cataract in our study was associated with the female sex, the urban region of habitation, lower level of high-density lipoproteins, higher diastolic blood pressure, lower level of education, higher stage of chronic kidney disease, more smoking package years, a lower prevalence of age-related macular degeneration and shorter axial length after adjusting for older age (Table 2). While many of these associations have also been found in previous investigations, it was interesting that in the multivariable analysis, a higher frequency of nuclear cataract was correlated with a lower prevalence of age-related macular degeneration. Since the present study, as a cross-sectional investigation, did not allow for conclusions to be drawn on causal relationships, a protective effect of nuclear cataract against the development of age-related macular degeneration may not be deduced from this finding. Interestingly, a higher prevalence of cortical cataract was associated with a lower prevalence of age-related macular degeneration in the multivariable analysis (Table 3).

Several limitations of our study should be mentioned. First, the quality of a population-based investigation markedly depends on the participation rate and the representativeness of the study population. With a participation rate of 80.5% of the eligible population, a major bias in the inclusion of participants of our study appears unlikely. The rural and urban study regions in the Russian republic of Bashkortostan are typical of the whole region of south Russia and the Volga Federal District in terms of demography, with a multiethnic and multicultural population structure including Muslim Turkic groups, such as Bashkirs and Tartars, geography with vast plains, and climate ranging from subcontinental climate to winter-cold wetland steppe climates. With respect to the ethnic background, the percentage of Russians was lower in our study region than in northwestern Russia and central Russia. To overcome this limitation, we examined the prevalence of cataract and cataract surgery in dependence on the ethnic background and did not detect significant associations between the ethnic background and the prevalence of cataract or cataract surgery. In addition, the distribution of age and sex in our study population was comparable to the results of the Russian census 2010^18,19^. Second, the group of individuals with available lens photographs for the assessment of cataract differed from the group without such photographs in age and sex. The younger age of individuals with cataract assessment may have led to an underestimation of the prevalence of cataract in the total study population. The prevalence of cataract and cataract-related MSVI in our study population was, however, relatively high and might have been even higher if more elderly individuals had been included in the study population. Third, we tested associations between the prevalence of cataract or pseudophakia and a whole panoply of ocular and systemic parameters, which might have led to falsely high significances in the univariate analyses. In full recognition of this potential weakness, the subsequent multivariate analyses assessed these associations with additionally taking into account interdependencies between the independent parameters. In addition, upon Bonferroni correction of the results of the multivariate analyses, the associations of the prevalence of previous cataract surgery with all parameters; the associations of nuclear cataract with all parameters except for serum concentration of high-density lipoproteins, diastolic blood pressure, level of education and axial length; the associations of cortical cataract with all parameters except for the prevalence of age-related macular degeneration; and the associations of subcapsular posterior cataract with age all remained statistically significant. The strengths of our study were the relatively large study population size and the relatively high number of ocular and systemic parameters examined in the investigation.

In conclusion, in this typical ethnically mixed population from Russia with an age of 40 + years, the prevalence of cataract in general and the prevalence of cataract-related MSVI and blindness were relatively high. Subsequently, the prevalence of previously performed cataract surgery was relatively low. In view of the aging of the Russian population, an increase in the surgical cataract rate in Russia is needed.
